# Skeletal Muscle Index Changes on Locoregional Treatment Application After FOLFIRINOX and Survival in Pancreatic Cancer

**DOI:** 10.1002/jcsm.13643

**Published:** 2024-11-23

**Authors:** Ji Hye Min, Jeong Il Yu, Seong Hyun Kim, Young Kon Kim, Kangpyo Kim, Hee Chul Park, Joon Oh Park, Jung Yong Hong, Kyu Taek Lee, Kwang Hyuck Lee, Jong Kyun Lee, Joo Kyung Park, Jin Ho Choi, Jin Seok Heo, In Woong Han, Hongbeom Kim, Sang Hyun Shin, So Jung Yoon, Sook‐young Woo

**Affiliations:** ^1^ Department of Radiology, Samsung Medical Center Sungkyunkwan University School of Medicine Seoul South Korea; ^2^ Department of Radiation Oncology, Samsung Medical Center Sungkyunkwan University School of Medicine Seoul South Korea; ^3^ Divisions of Hematology‐Oncology, Department of Medicine, Samsung Medical Center Sungkyunkwan University School of Medicine Seoul South Korea; ^4^ Divisions of Gastroenterology, Department of Medicine, Samsung Medical Center Sungkyunkwan University School of Medicine Seoul South Korea; ^5^ Department of Surgery, Samsung Medical Center Sungkyunkwan University School of Medicine Seoul South Korea; ^6^ Biomedical Statistics Center, Research Institute for Future Medicine Samsung Medical Center Seoul South Korea

**Keywords:** FOLFIRINOX, locoregional treatments, pancreatic ductal adenocarcinoma, sarcopenia, skeletal‐muscle‐index changes

## Abstract

**Background:**

Patients with borderline resectable (BR) or locally advanced pancreatic cancer (LAPC) require complex management strategies. This study evaluated the prognostic significance of the perichemotherapy skeletal muscle index (SMI) and carbohydrate antigen 19‐9 (CA 19‐9) in patients with BRPC or LAPC treated with FOLFIRINOX.

**Methods:**

We retrospectively evaluated 227 patients with BR or LAPC who received at least four cycles of chemotherapy between 2015 and 2020. We analysed chemotherapy response, changes in SMI (ΔSMI, %) on computed tomography (CT) and CA19‐9 to determine their impact on progression‐free survival (PFS) and overall survival (OS). After the early application of loco‐regional treatments (LRT) within 3 months after completing four cycles of chemotherapy, the outcomes were compared between ΔSMI and CA19‐9 subgroups.

**Results:**

Among 227 patients (median age, 60 years; 124 [54.6%] male) with 97 BR and 130 LAPC, 50.7% showed partial response (PR) to chemotherapy, 44.5% showed stable disease and 4.8% showed progressive disease (PD). Post‐chemotherapy CA19‐9 levels were normalized in 41.0% of patients. The high and low ΔSMI groups (based on the gender‐specific cut‐off of −8.6% for males and −2.9% for females) comprised 114 (50.2%) and 113 (49.8%) patients, respectively. The high ΔSMI group had poorer survival rates than the low ΔSMI group in both PFS (HR = 1.32, *p* = 0.05) and OS (HR = 1.74, *p* = 0.001). Multivariable analysis showed that ΔSMI (high vs. low; PFS, HR = 1.39, *p* = 0.03; OS, HR = 1.82, *p* < 0.001) and post‐chemotherapy response (PD vs. PR/SD; PFS, HR = 18.69, *p* < 0.001; OS, HR = 6.19, *p* < 0.001) were independently associated with both PFS and OS. Additionally, the post‐chemotherapy CA19‐9 (≥ 37 vs. < 37; HR = 1.48, *p* = 0.01) was an independent predictor for PFS. Early application of LRT after chemotherapy significantly improved PFS and OS in both ΔSMI groups (all *p* < 0.05). However, it was not beneficial in the group with high ΔSMI and post‐chemotherapy CA19‐9 ≥ 37 (PFS, *p* = 0.39 and OS, *p* = 0.33).

**Conclusions:**

Progressive sarcopenic deterioration after four cycles of chemotherapy was associated with poor survival outcomes in patients with BR or LAPC after FOLFIRINOX. We also investigated the optimal clinical setting for the early application LRTs using the ΔSMI and post‐chemotherapy CA 19‐9.

## 1 Introduction

Pancreatic cancer is one of the most challenging malignancies with a high mortality rate and is predicted to become an increasingly important cause of death in the future [1]. Despite considerable improvement in understanding of the biology of pancreatic cancer and treatment modalities, including systemic agents and surgical/radiation techniques, the outcomes of pancreatic ductal adenocarcinoma (PDAC) remain poor, with a 5‐year survival rate of less than 10%, emphasizing the significance of optimizing treatment strategies [2, 3]. The approach to PDAC treatment is largely based on its resectability at the time of diagnosis. A key factor for long‐term survival is complete surgical resection; however, only 15%–20% of PDACs are resectable [4]. Furthermore, over half of these patients will develop distant metastases after resection, highlighting the aggressive nature of PDAC [5].

Given these circumstances, neoadjuvant therapy has emerged as a crucial treatment, aiming to reduce micrometastasis and enhance the tumour resectability of PDAC, even in initially resectable cases [6]. In particular, the introduction of combination chemotherapy consisting of folinic acid, 5‐fluorouracil, irinotecan and oxaliplatin (FOLFIRINOX) has marked a paradigm shift in treatment [7]. The FOLFIRINOX regimen has outperformed conventional gemcitabine‐based regimens and is recommended as the most preferred first line regimen in patients with borderline resectable pancreatic cancer (BRPC) and locally advanced pancreatic cancer (LAPC) [6, 8]. Thereafter, a multidisciplinary approach for BR or LAPC, which integrates surgical resection and/or radiation therapy (RT) after neoadjuvant or induction FOLFIRINOX, is advocated whenever possible. Although the benefits of multimodal approaches in BR/LAPC patients seem clear, optimal treatment remains a challenge, especially concerning the timing and selection of candidates for loco‐regional treatments (LRT) after FOLFIRINOX. In particular, given the unreliability of radiologic imaging for reassessment of resectability, reassessments primarily rely on the level of carbohydrate antigen 19‐9 (CA 19‐9) and positron emission tomography‐compute tomography (PET‐CT) findings known to reflect the tumour biology; however, the status of the patient's condition, which could in itself potentially reflect treatment response, sustainability of further treatment and host immune status, has been insufficiently considered.

Previous studies have evaluated the association between sarcopenia and treatment outcome in many cancers, including PDAC [9, 10, 11]. However, most studies have utilized sarcopenia data from a single time point, usually before treatment initiation. Notably, no study has evaluated the role of sequential changes in skeletal muscle index (SMI) on post‐treatment prognosis of BR or LAPC following FOLFIRINOX treatment. Therefore, we evaluated the prognostic significance of the peri‐chemotherapy skeletal muscle index (ΔSMI, %) and CA 19‐9 in BRPC or LAPC patients treated with FOLFIRINOX. Furthermore, we analysed the optimal clinical setting to maximize the effect of subsequent LRT, such as surgical resection and/or RT after four cycles of FOLFIRINOX treatment.

## 2 Methods

### 2.1 Patients

This study was approved by the International Review Board of Samsung Medical Center (IRB no. SMC 2022‐05‐139). The requirement for written informed consent was waived owing to the retrospective nature of the study. We retrospectively searched Samsung Medical Center databases between December 2015 and December 2020 to identify patients with PDAC. The inclusion criteria were as follows: (a) patients with PDAC pathologically proven through endoscopic biopsy; (b) patients with PDAC classified as either BRPC or LAPC according to the National Comprehensive Cancer Network (NCCN) guidelines (Supporting Information S1) [12]; (c) patients who planned FOLFIRINOX (started within 2 months of diagnosis), with the intention to complete at least four cycles; (d) patients who underwent contrast‐enhanced computed tomography (CT) at baseline and after four cycles (or the final cycle if fewer than four) of FOLFIRINOX. The exclusion criteria were as follows: (a) patients with distant metastasis (*n* = 223); (b) history of other malignancies (*n =* 69); (c) patients who did not receive all four cycles of chemotherapy (*n* = 21); (d) unavailable baseline or follow‐up CT (*n* = 10); (e) incomplete data for the analysis (*n* = 10); (f) resectable PDAC at diagnosis (*n* = 2). A flowchart of the study population is presented (Supporting Information S2).

### 2.2 Pretreatment Evaluation

Medical history, physical examinations and laboratory tests were conducted for pretreatment assessment. Baseline radiologic staging was performed using chest CT, dynamic contrast‐enhanced abdominal CT, magnetic resonance imaging (MRI) and PET‐CT. Resectability was determined using diagnostic CT and/or MRI according to the NCCN guidelines.

### 2.3 Data Collection and Definition

Age, sex, height, weight, body mass index (BMI), tumour location (head/neck and body/tail), tumour size, clinical stage at diagnosis (eighth edition The American Joint Committee on Cancer staging manual), initial resectability and type of LRT after chemotherapy were collected [13]. The chemotherapy response (partial response [PR], stable disease [SD] and progressive disease [PD]) after four cycles of chemotherapy was also collected.

Serial changes in CA 19‐9 and the systemic inflammation markers (SIMs), such as neutrophil‐to‐lymphocyte ratio (NLR), lymphocyte‐to‐monocyte ratio (NMR) and platelet‐to‐lymphocyte ratio (PLR), were evaluated pre‐chemotherapy and after four cycles of chemotherapy. The CA19‐9 categories were categorized: baseline (< 500 U/mL vs. ≥ 500 U/mL) and post‐chemotherapy (< 37 U/mL [normalization either normal or elevated baseline CA 19‐9] vs. ≥ 37 U/mL).

The total abdominal muscle area (TMA) was measured on CT images pre‐chemotherapy and after four cycles of chemotherapy, as described in previous studies [14, 15]. The skeletal muscle index (SMI) was calculated as TMA (cm^2^)/height (m^2^) (Supporting Information S3). Sarcopenia was defined by an SMI < 42.2 cm^2^/m^2^ for men and < 33.9 cm^2^/m^2^ for women [16, 17]. The peri‐chemotherapy SMI changes (ΔSMI, %) were then calculated for each patient by comparing the SMI values pre‐ and post‐four cycles of chemotherapy using the formula [14]: ΔSMI (%) = ([SMI on pre‐chemotherapy CT − SMI on post‐four cycles of chemotherapy CT]/SMI on pre‐chemotherapy CT) × 100. Patients were classified into two groups (high vs. low) based on the gender‐specific median SMI values.

### 2.4 Treatment and Follow‐Up Evaluation

The FOLFIRINOX treatment for PDAC was administered following a comprehensive multidisciplinary team discussion [18]. Further details on this protocol are provided in Supporting Information S4. Patients were re‐evaluated with CT scans of the chest, abdomen and pelvis and tumour markers after every four cycles of FOLFIRINOX. The chemotherapy response was evaluated according to the revised Response Evaluation Criteria in Solid Tumours (RECIST V.1.1) [19]. If surgical resection was deemed feasible, additional abdominal MRI and PET‐CT images were obtained to assess tumour resectability and the existence of distant metastatic lesions. The decision on whether and which treatment to pursue was made through multidisciplinary consultation. We defined ‘early application of LRT’ as LRT applied within 3 months after completing the four cycles of chemotherapy.

### 2.5 Outcome

The primary treatment outcome was progression‐free survival (PFS), and the secondary outcome was overall survival (OS). The PFS was calculated from the initiation date of FOLFIRINOX to the date of disease progression, death or the last follow‐up visit, whichever came first. The OS was calculated from the initiation date of FOLFIRINOX to the date of death or the last follow‐up visit, whichever came first. The patients were followed‐up until death or 31 May 2022.

### 2.6 Statistical Analysis

Patient and treatment characteristics were summarized using mean with standard deviation (SD) or median with interquartile range (IQR), range (minimum, maximum) for continuous variables and frequency with percentage for categorical variables. Perichemotherapy changes in CA 19‐9, SMI, sarcopenia and SIMs were compared using Wilcoxon signed rank tests for continuous data and McNemar tests for categorical variables.

The OS and PFS were estimated using the Kaplan–Meier method and compared using the log‐rank test. The associations between the variables and outcomes were analysed using a Cox proportional hazards model. Schoenfeld residual tests were performed for the proportional hazards assumption check. There was no variables with violating proportional hazards assumption. A multivariable model was built using variables with a *p*‐value ≤ 0.1 in the univariable analysis. Factors showing multicollinearity were excluded from the multivariable analyses. Baseline NLR, LMR and PLR variables with skewed distribution were transformed using natural log after adding 1 due to zero values ΔNLR, ΔLLR and ΔPLR (%) variables with skewed distribution were transformed using natural log after adding 100 due to zero values.

To identify the optimal clinical conditions after four chemotherapy cycles in which subsequent early application of LRTs could achieve maximum therapeutic efficacy, we performed log rank test for ΔSMI and post‐chemotherapy CA 19‐9 in conditional subgroups, while intentionally excluding the chemotherapy response (a well‐established factor for subsequent treatment decisions). Statistical analysis was conducted using SAS version 9.4 (SAS Institute, Cary, NC, USA) and R 4.1.0 (Vienna, Austria; http://www.R‐project.org).

## 3 Results

### 3.1 Patient and Tumour Characteristics

The baseline characteristics of the 227 patients (median age 60 years; IQR 56, 67 years; 54.6% male and 45.4% female) are summarized in Table 1. The mean BMI was 23.0 ± 2.8. The PDACs were located in the pancreatic head/neck (*n* = 148, 65.2%) and the body/tail (*n* = 79, 34.8%). The median tumour size at diagnosis was 3.1 cm. Based on the NCCN guideline, 97 (42.7%) and 130 (57.3%) patients were classified into the BRPC and LAPC groups, respectively.

**TABLE 1:** Patients and treatment characteristics.

	Total (*n* = 227)
*Patients*	
Age (years)^a^	60 (56, 67)
Sex, Male	124 (54.6)
Height (m)	162.3 ± 8.8 (range, 143.4–185.4)
Weight (kg)	59.5 ± 12.8 (range, 34.8–88.5)
BMI	23.0 ± 2.8 (range, 15.9–31.9)
Tumour location	
Head/neck	148 (65.2)
Body/tail	79 (34.8)
Tumour size (cm)^a^	3.1 (2.5, 4)
T stage	
T1	17 (7.5)
T2	90 (39.6)
T3	26 (11.5)
T4	94 (41.4)
N stage	
N0	154 (67.8)
N1	68 (30.0)
N2	5 (2.2)
Initial resectability	
BR	97 (42.7)
LA	130 (57.3)
Chemotherapy response^b^	
PR	115 (50.7)
SD	101 (44.5)
PD	11 (4.8)
Subsequent LRTs	133 (58.6)
Surgery only	52 (22.9)
Radiation only	57 (25.1)
Surgery and radiation	24 (10.6)
Early application of LRTs within 3 months after completing 4 cycles of chemotherapy	70 (30.8)
Surgery	54 (23.8)
Radiation	16 (7.1)

*Note:* Data are expressed as *n* (%) or mean ± standard deviation (range, minimum–maximum), unless otherwise indicated.

Abbreviations: BMI, body mass index; BR, borderline resectable; LA, locally advanced; LRT, loco‐regional treatment; PD, progressive disease; PR, partial response; SD, stable disease.

^a^ Median (interquartile range).

^b^ At the time of completion of 4 cycles of chemotherapy.

The treatment response after four cycles of FOLFIRINOX was as follows: 115 (50.7%), 101 (44.5%) and 11 (4.8%) for PR, SD and PD, respectively. Regarding subsequent LRT, 133 patients (58.6%) underwent LRT, ultimately including surgery (*n* = 52, 22.9%), RT (*n* = 57, 25.1%) or both (*n* = 24, 10.6%). The median times from the completion of four cycles of chemotherapy to surgery and RT were 2.3 months (IQR: 1.5, 5 months) and 5.8 months (IQR: 2.1, 7.1 months), respectively. Among these, 70 patients (30.8%) received early application of LRT defined as the application of LRT within 3 months after completing four cycles of chemotherapy. Among these, 54 (23.8%) patients underwent surgery and 16 (7.1%) received RT.

### 3.2 Perichemotherapy Changes

Following the completion of four cycles of chemotherapy, the median CA19‐9 levels decreased from 136.0 U/mL (IQR: 36.1, 629.4 U/mL; range: 1.2–28594.3) to 60.8 U/mL (IQR: 17.5, 259.1 U/mL; range: 1.2–8168.8) (Table 2). At baseline, 72 (31.7%) patients presented with CA19‐9 levels ≥ 500 U/mL. After chemotherapy, 93 (41.0%) had normalized CA 19‐9 (< 37 U/mL), and 134 (59.0%) had elevated CA 19‐9 level (≥ 37 U/mL).

**TABLE 2:** Perichemotherapy changes.

	Baseline	PostCTx	*p*
Laboratory findings			
CA19‐9 (U/mL)	136.0 (36.1, 629.4) [range 1.2–28594.3]	60.8 (17.5, 259.1) [range 1.2–8168.8]	< 0.001
Baseline CA19‐9 category^a^			< 0.001
< 500 U/mL	155 (68.3)	191 (84.1)	
≥ 500 U/mL	72 (31.7)	36 (15.9)	
Post CTx CA19‐9 category^a^			< 0.001
< 37 U/mL	58 (25.6)	93 (41.0)	
≥ 37 U/mL	169 (74.4)	134 (59.0)	
SMI, total	43.8 (40.1, 51.5) [range 27.6–70.3]	41.3 (36.9, 46.9) [range 21.9–68.6]	< 0.001
SMI, male	49.1 (44.6, 54.9) [range 34.9–70.3]	45.2 (40.6, 50.2) [range 31.6–68.6]	< 0.001
SMI, female	40.7 (36.5, 42.6) [range 27.6–58.0]	38.0 (34.8, 41.6) [range 21.9–53.2]	< 0.001
Sarcopenia, total^b^	30 (13.2)	61 (26.9)	< 0.001
Sarcopenia, male (*n* = 124)	17 (13.7)	42 (33.9)	< 0.001
Sarcopenia, female (*n* = 103)	13 (12.6)	19 (18.4)	0.109
NLR	2.1 (1.6, 2.9)	1.1 (0.7, 1.8)	< 0.001
LMR	4.0 (3.0, 5.3)	3.0 (2.4, 4.0)	< 0.001
PLR	127.5 (101.6, 168.1)	108.1 (76.0, 143.7)	< 0.001
Perichemotherapy changes			
Δ SMI (%), total^c^	−6.1 (−11.9, −0.4)		
Δ SMI (%), male^c^	−8.6 (−14.5, −3.1)		
Δ SMI (%), female^c^	−2.9 (−8.8, 1.7)		
Δ SMI (%) category^c^			
High SMI group	114 (50.2)		
Low SMI group	113 (49.8)		
Δ NLR (%)	−43.7 (−66.7, −9.6)		
Δ LMR (%)	−24.3 (−42.7, 5.8)		
Δ PLR (%)	−15.4 (−41.3, 13.2)		

*Note:* Data are expressed as *n* (%) or median (interquartile range) [range, minimum‐maximum], unless otherwise indicated. Continuous variables were analysed using Wilcoxon's signed rank test. Categorical variables were analysed using McNemar test.

Abbreviations: CA19‐9, carbohydrate antigen 19‐9; CTx, chemotherapy; LMR, lymphocyte‐to‐monocyte ratio; NLR, neutrophil‐to‐lymphocyte ratio; PLR, platelet‐to‐lymphocyte ratio; SMI, skeletal muscle index.

^a^ The CA19‐9 categories were categorized: baseline (< 500 U/mL vs. ≥ 500 U/mL) and post‐chemotherapy (< 37 U/mL [normalization either normal or elevated baseline CA 19‐9] vs. ≥ 37 U/mL).

^b^ The sarcopenia was defined by an SMI < 42.2 cm^2^/m^2^ for men and < 33.9 cm^2^/m^2^ for women.

^c^ The peri‐chemotherapy SMI changes (ΔSMI, %) were then calculated for each patient by comparing the pre and post 4 cycles of chemotherapy SMI values using the formula: ΔSMI (%) = ([SMI at pre‐chemotherapy CT − SMI at post 4 cycles of chemotherapy CT]/SMI at pre‐chemotherapy CT) × 100. Patients were classified into two groups (high vs. low) based on the gender‐specific median SMI values.

The median SMI values decreased from 43.8 (baseline) to 41.3 (post‐chemotherapy) (*p* < 0.001). Median ΔSMI values of male and female patients were −8.6% and −2.9%, respectively. Using the gender‐specific median ΔSMI (%) cut‐offs, 114 (50.2%) and 113 (49.8%) patients were classified into the high and low ΔSMI groups, respectively. The characteristics of high and low ΔSMI groups are provided in Supporting Information S5.

After four cycles of chemotherapy, the median values of NLR, LMR and PLR decreased (2.1 to 1.1, 4.0 to 3.0 and 127.5 to 108.1, respectively; all *p*‐values < 0.001). The median values of ΔNLR (%), ΔLMR (%) and ΔPLR (%) were −43.7%, −24.3% and −15.4%, respectively.

### 3.3 Survival Outcomes

The median follow‐up period was 20 months (range, 3–73 months). At the time of analysis, 143 (63.0%) of 227 patients had died, and 55 (24.2%) have shown disease progression without death. The median PFS and OS of all patients were 13.4 months (95% confidence interval [CI], 12.0–15.2) and 24.2 months (95% CI, 20.3–29.8), respectively.

### 3.4 Prognostic Factor Analysis

In the multivariable analysis (Table 3), the post‐chemotherapy CA19‐9 (≥ 37 vs. < 37; hazard ratio [HR], 1.48; 95% CI, 1.09–2.02; *p* = 0.01), ΔCA 19‐9 (≥ −49% vs. < −49%; HR, 1.37; 95% CI, 1.03–1.84; *p =* 0.03), ΔSMI (high vs. low; HR 1.39; 95% CI, 1.04–1.87; *p* = 0.03) and post‐chemotherapy response (PD vs. PR/SD; HR, 18.69; 95% CI, 9.08–38.46; *p* < 0.001) were independently correlated with PFS. In addition, the resectability (LA vs. BR; HR, 1.53; 95% CI, 1.03–2.28; *p* = 0.04), baseline sarcopenia (HR 2.13, 95% CI, 1.34–3.39; *p* = 0.001), ΔSMI (high vs. low; HR 1.82, 95% CI, 1.29–2.57; *p* < 0.001) and post‐chemotherapy response (PD vs. PR/SD; HR, 6.19; 95% CI, 3.05–12.60; *p* < 0.001) were independently correlated with the OS.

**TABLE 3:** Prognostic factor analysis of progression free survival and overall survival.

Variables	Progression‐free survival				Overall survival			
	Univariable		Multivariable		Univariable		Multivariable	
	HR (95% CI)	*p*	HR (95% CI)	*p*	HR (95% CI)	*p*	HR (95% CI)	*p*
Sex, female [male]	1.08 (0.82–1.43)	0.59			0.93 (0.67–1.30)	0.68		
Age (years) ≥ 65 [< 65]	1.25 (0.93–1.70)	0.15			1.34 (0.94–1.91)	0.10		
BMI ≥ 25 [< 25]	1.11 (0.78–1.57)	0.57			0.91 (0.59–1.30)	0.91		
Tumour location, head/neck [body/tail]	1.18 (0.88–1.58)	0.27			1.11 (0.78–1.57)	0.57		
Resectability, LA [BR]	1.62 (1.21–2.16)	< 0.001	1.08 (0.77–1.52)	0.65	1.92 (1.36–2.72)	< 0.001	1.53 (1.03–2.28)	0.04
Baseline CA19‐9 ≥ 500 (< 500)	1.24 (0.92–1.67)	0.16			1.76 (1.26–2.46)	0.003	1.17 (0.78–1.76)	0.46
Post‐chemotherapy CA19‐9 ≥ 37 (< 37)	1.62 (1.21–2.15)	0.001	1.48 (1.09–2.02)	0.01	1.88 (1.33–2.67)	0.001	1.39 (0.90–2.15)	0.14
ΔCA 19‐9 ≥ −49% (< −49%)^a^	1.41 (1.07–1.87)	0.02	1.37 (1.03–1.84)	0.03	1.57 (1.13–2.19)	0.02		
Baseline sarcopenia, yes [no]^b^	1.51 (1.01–2.25)	0.05	1.35 (0.88–2.09)	0.17	1.83 (1.18–2.84)	0.007	2.13 (1.34–3.39)	0.001
Post‐chemotherapy sarcopenia, yes [no]^a^, ^b^	1.39 (1.01–1.89)	0.04			1.65 (1.16–2.35)	0.006		
ΔSMI, high group [low group]^c^	1.32 (1.00–1.74)	0.05	1.39 (1.04–1.87)	0.03	1.74 (1.25–2.43)	0.001	1.82 (1.29–2.57)	< 0.001
Baseline NLR	1.21 (0.78–1.87)	0.40			1.09 (0.67–1.78)	0.73		
Baseline LMR	0.62 (0.39–0.99)	0.05	0.85 (0.47–1.57)	0.61	0.71 (0.44–1.15)	0.17		
Base PLR	1.39 (0.98–1.98)	0.07	1.13 (0.76–1.69)	0.54	1.39 (0.93–2.08)	0.10		
ΔNLR %	1.06 (0.88–1.27)	0.55			1.12 (0.90–1.38)	0.31		
ΔLMR %	1.24 (0.98–1.58)	0.07	1.18 (0.87–1.61)	0.30	1.27 (0.96–1.68)	0.10		
ΔPLR %	0.92 (0.68–1.23)	0.56			0.94 (0.67–1.32)	0.71		
Post‐chemotherapy response, PD [PR/SD]	16.38 (8.51–31.53)	< 0.001	18.69 (9.08–38.46)	< 0.001	5.94 (2.97–11.89)	< 0.001	6.19 (3.05–12.60)	< 0.001
Early application of LRTs, yes [no]	0.52 (0.38–0.72)	< 0.001	0.70 (0.48–1.01)	0.05	0.48 (0.33–0.71)	< 0.001	0.73 (0.47–1.14)	0.17

*Note:* The reference category for each variable is shown in the square brackets in the first column. All statistical analyses of prognostic factors were conducted using the Cox proportional hazards model. Baseline NLR, LMR and PLR variables with skewed distribution were transformed using natural log after adding 1 due to zero values. ΔNLR, ΔLLR and ΔPLR variables with skewed distribution were transformed using natural log after adding 100 due to zero values.

Abbreviations: BMI, body mass index; BR, borderline resectable; CI, confidence interval; HR, hazard ratio; LA, locally advanced; LMR, lymphocyte‐to‐monocyte ratio; LRT, loco‐regional treatment; NLR, neutrophil‐to‐lymphocyte ratio; PD, progressive disease; PLR, platelet‐to‐lymphocyte ratio; PostCTx, post‐chemotherapy; PR, partial response; SD, stable disease; SMI, skeletal muscle index.

^a^ Factors showing multicollinearity were excluded from the multivariable analyses.

^b^ The sarcopenia was defined by an SMI < 42.2 cm^2^/m^2^ for men and < 33.9 cm^2^/m^2^ for women.

^c^ High and low groups of ΔSMI were divided based on the gender‐specific median value of SMI change as the cut‐off of −8.6% for males and −2.9% for females.

### 3.5 Survival Outcomes According to ΔSMI Group

**FIGURE 1:**
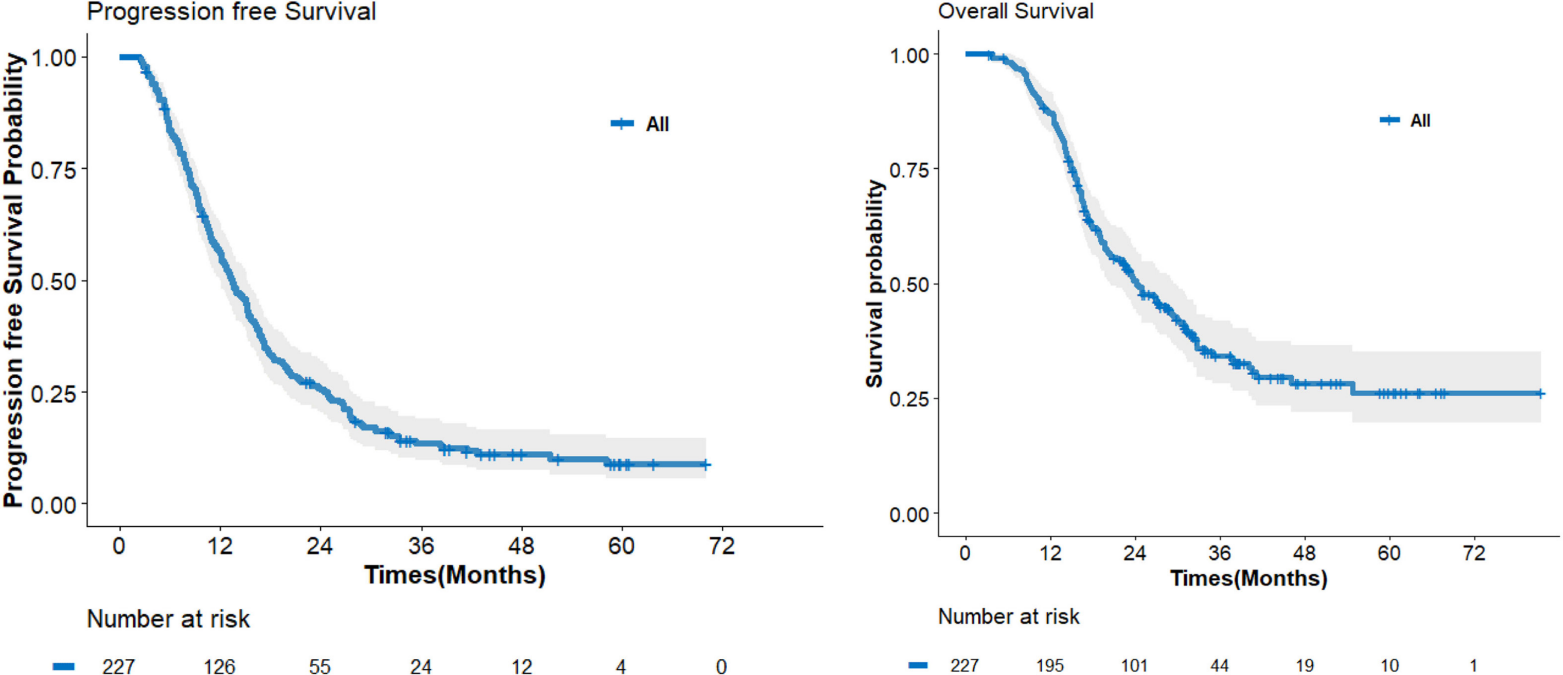
Survival outcomes for the all patients.

**FIGURE 2:**
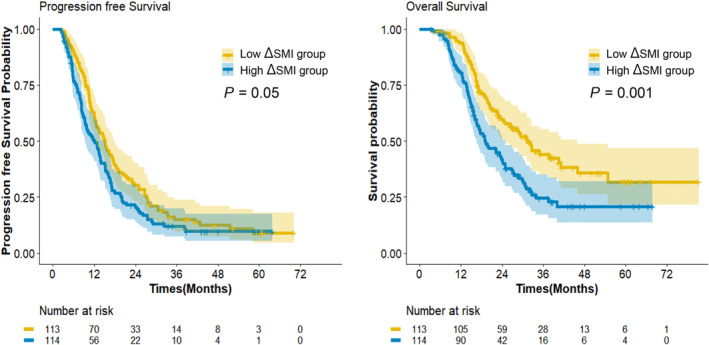
Survival outcomes according to the changes of skeletal muscle index (ΔSMI) % group. SMI, skeletal muscle index.

In all patients, the 1‐, 2‐, 3‐, and 5‐year PFS and OS rates were 56.0%, 25.5%, 13.6%, 9.0% and 87.1%, 50.7%, 34.3%, 26.2%, respectively (Figure 1). The high ΔSMI group showed poorer PFS and OS rates than the low ΔSMI group (PFS; HR, 1.32; 95% CI 1.00–1.74; *p* = 0.05 and OS; HR, 1.74; 95% CI 1.25–2.43; *p* = 0.001) (Figure 2). The details of the 1‐, 2‐, 3‐ and 5‐year survival rates of the two groups based on ΔSMI are provided in Table 4.

**TABLE 4:** Survival outcomes of all patients and subgroup analysis for ΔSMI and post‐chemotherapy CA 19‐9.

	Progression‐free survival					Overall survival				
	1‐year	2‐year	3‐year	5‐year	*p*	1‐year	2‐year	3‐year	5‐year	*p*
All	56.0	25.5	13.6	9.0		87.1	50.7	34.3	26.2	
ΔSMI group					0.05					0.001
Low	61.8	30.4	15.1	9.1		93.8	59.6	44.0	31.7	
High	50.1	20.5	12.1	9.7		80.5	41.8	24.5	20.8	
Subgroup (ΔSMI)										
Low ΔSMI group					0.002					0.007
With LRT	64.1	45.9	28.8	23.0		94.9	71.6	61.4	51.9	
Without LRT	60.6	22.0	7.9	3.9		93.2	53.0	33.6	25.7	
High ΔSMI group					0.02					0.02
With LRT	73.4	30.0	23.4	15.6		87.9	59.5	43.6	32.3	
Without LRT	41.5	17.1	7.7	7.7		78.0	35.1	16.3	16.3	
Subgroup (ΔSMI and elevated post‐chemotherapy CA 19‐9)^a^										
Group 1 (no risk factor)					0.04					0.22
With LRT	68.0	48.0	30.9	20.6		92.0	72.0	60.9	52.2	
Without LRT	69.4	27.8	6.9	3.5		90.0	65.6	44.5	28.6	
Group 2 (Any one risk factor)					0.003					0.010
With LRT	73.3	42.9	32.1	24.1		96.7	73.0	57.7	46.1	
Without LRT	52.3	21.5	8.7	5.8		90.6	44.7	27.3	13.7	
Group 3 (two risk factors)					0.39					0.33
With LRT	57.4	14.1	7.2	7.2		79.4	40.6	30.5	20.3	
Without LRT	39.3	13.1	7.5	7.5		77.0	30.9	11.2	11.2	

*Note:* Progression free survival rates and overall survival rates calculated using Kaplan–Meier method, and *p* value calculated using log rank test.

Abbreviations: LRT, local regional treatment; SMI, skeletal muscle index.

^a^ We considered the high ΔSMI and elevated post‐chemotherapy CA 19‐9 (≥ 37 U/mL) as two risk factors for poor survival outcomes. Group 1 consisted of patients with no risk factors, Group 2 included those with one risk factor and Group 3 comprised patients with both risk factors.

### 3.6 Survival Outcomes After Early Application of LRT According to ΔSMI Group

**FIGURE 3:**
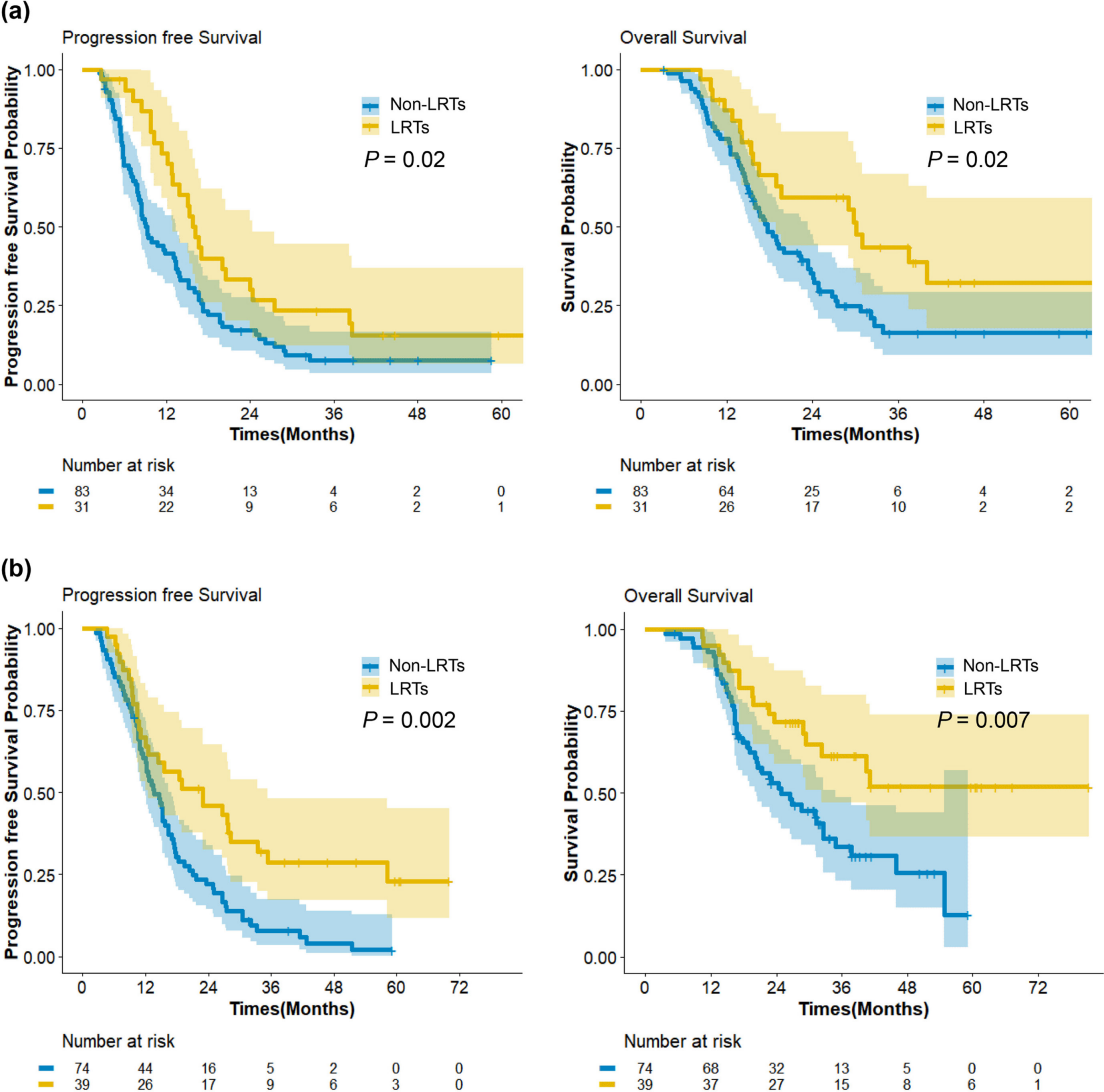
Survival outcomes after application of early loco‐regional treatment according to ΔSMI % group. (a) Progression free survival and overall survival in high ΔSMI % group. (b) Progression free survival and overall survival in low ΔSMI % group. LRT, loco‐regional treatment; SMI, skeletal muscle index.

Early application of LRTs after four chemotherapy cycles resulted in better PFS and OS in both high and low ΔSMI groups (all *p* < 0.05) (Figure 3). The details of the 1‐, 2‐, 3‐, and 5‐year survival rates of both groups are provided in Table 4. The characteristics according to the time of LRTs after completing 4 cycles of FOLFIRINOX are provided in Supporting Information S6.

### 3.7 Survival Outcomes After Early Application of LRT According to ΔSMI and Post‐Chemotherapy CA 19‐9 Subgroup

**FIGURE 4:**
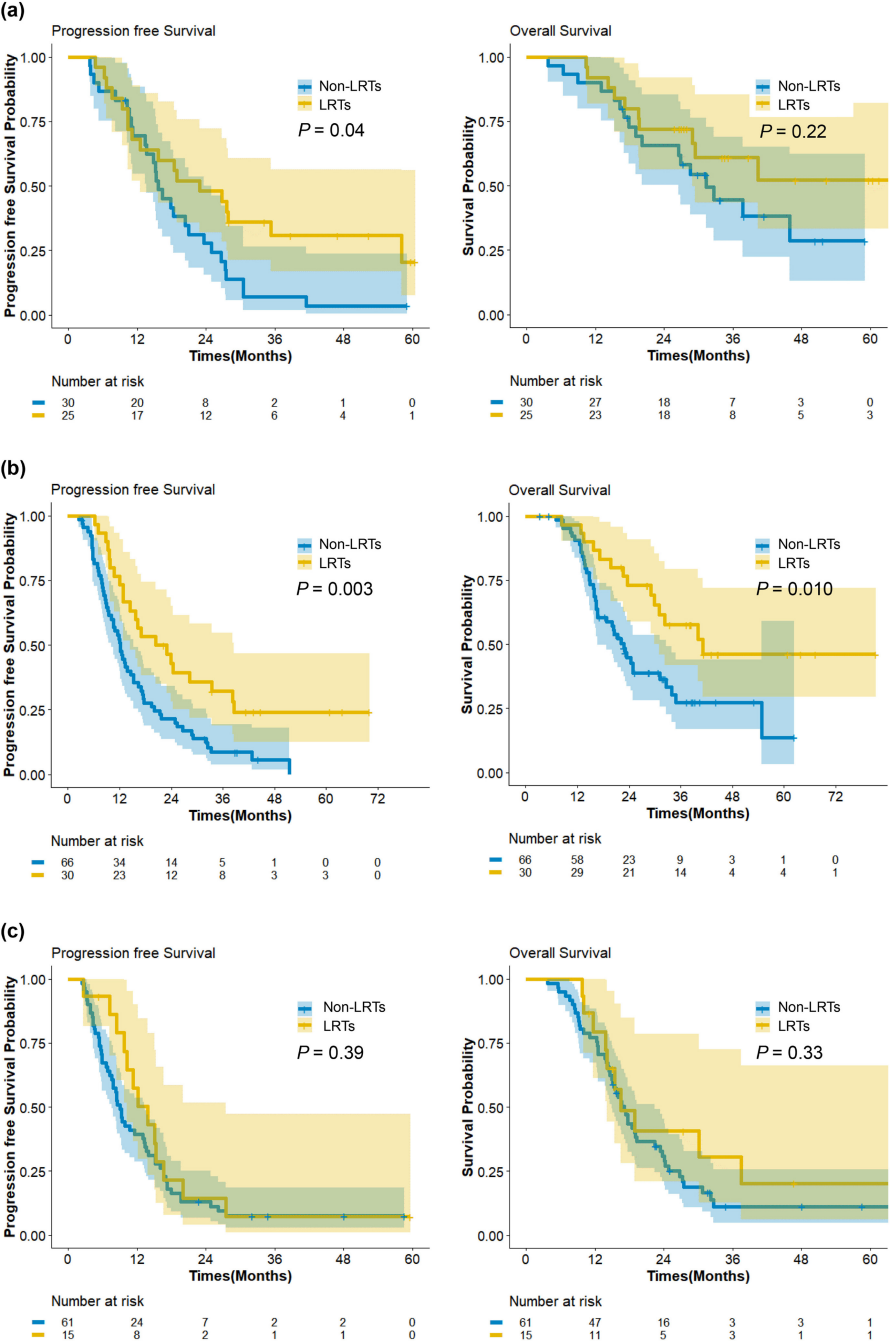
Survival outcomes after application of early loco‐regional treatment according to ΔSMI and postCTx CA 19‐9 subgroup. (a) Progression free survival and overall survival in group 1 (no risk factor). (b) Progression free survival and overall survival in group 2 (any one risk factor). (c) Progression free survival and overall survival in group 3 (two risk factors). LRT, loco‐regional treatment; SMI, skeletal muscle index.

We considered high ΔSMI and elevated post‐chemotherapy CA 19‐9 (≥37 U/mL) as two risk factors for poor survival outcomes (Table 4). Group 1 consisted of patients with no risk factors, Group 2 included those with one risk factor and Group 3 comprised patients with both risk factors. The Kaplan–Meier curves were calculated for the effects of the combination of ΔSMI and post‐chemotherapy CA 19‐9 on survival outcomes in the context of early application of LRTs. If both risk factors were present on completion of four cycles of chemotherapy (Group 3), early application of LRT did not benefit either PFS (*p* = 0.39) or OS (*p* = 0.33). For patients with no risk factor or one risk factor (Group 1 or 2), early application of LRT was beneficial for PFS (Figure 4). The characteristics according to the group of ΔSMI and post‐chemotherapy CA 19‐9 are provided in Supporting Information S7.

## 4 Discussion

In the present study, we evaluated the prognostic and predictive potential of perichemotherapy ΔSMI and CA 19‐9 in patients with BRPC or LAPC treated with FOLFIRINOX. The ΔSMI (high vs. low) and post‐chemotherapy response (PD vs. PR/SD) were independently associated with PFS and OS. Additionally, the post‐chemotherapy CA19‐9 (≥ 37 vs. < 37) was an independent predictor for PFS. Regarding the early application of LRT within 3 months after four cycles of chemotherapy, both high and low SMI groups showed a survival benefit from LRT. However, the trends for the benefit of early application of LRT were not consistent in patients with high ΔSMI and post‐chemotherapy CA19‐9 levels. In this clinical scenario, maintaining or modifying chemotherapy would be more reasonable than hastening LRT.

Our study aligns with previous research highlighting the effectiveness of adjuvant and neoadjuvant treatments in improving oncologic outcomes of pancreatic cancer [6, 7, 8]. FOLFIRINOX has become the preferred systemic therapy for BRPC/LAPC as well as metastatic PDAC, especially in patients with good performance status. The application of LRT, particularly surgery, is notably linked to the potential for improved treatment outcomes and, in some cases, achieving a cure [20, 21, 22]. Although the benefits of multimodal approaches in managing BRPC/LAPC are apparent, challenges remain in determining the optimal timing and candidate selection for LRT after FOLFIRINOX treatment. The heterogeneity of patient cohorts and varied treatment combinations in previous studies complicate this decision‐making process. Moreover, alternative treatments such as RT still lack robust supporting evidence [6]. Therefore, our study focused on the potential implications of ΔSMI and CA 19‐9 as markers for predicting responses and guiding decisions regarding the application of LRT after FOLFIRINOX therapy.

Recent studies have established sarcopenia as a negative prognostic factor in pancreatic cancer, particularly regarding postoperative complications and recovery [9, 10, 11]. Additionally, previous research has shown that surgery‐induced muscle loss following major abdominal surgery can impact on 1‐year oncological survival [23, 24]. The correlation between sarcopenia and immune suppression implies that aggressive surgical interventions in patients with sarcopenia may lead to poorer outcomes [10]. Our findings reflect this, showing limited benefits of LRT in certain patient subsets, despite being feasible. Furthermore, the clinical significance of SMI changes on patient outcomes is widely recognized in various cancers, indicating its potential as a predictive marker for treatment outcomes [25, 26, 27, 28]. These insights advocate for incorporating SMI changes and CA 19‐9 assessments into treatment planning. Our findings indicate that early application of LRT may not be beneficial, particularly in patients with high ΔSMI and post‐chemotherapy CA 19‐9, despite their clinical eligibility for LRT. Identifying high‐risk patients allows for more personalized treatment strategies. The findings raise the possibility that continued or modified chemotherapy might be more beneficial than hastening LRT in certain patient subsets, especially those showing peri‐chemotherapy sarcopenic deterioration, thus potentially improving overall treatment efficacy and patient outcomes in pancreatic cancer.

The utility of CA 19‐9 and PET‐CT in pancreatic cancer treatment decisions during adjuvant treatment is well‐studied; however, limitations exist [29, 30]. Although CA 19‐9 is a well‐established marker, there is no consensus on post‐chemotherapy cut‐off values for decision‐making. PET‐CT, while useful in assessing tumour biological activity, is limited by high costs, limited availability, and the need for specialized interpretation. The NCCN acknowledges the value of PET‐CT in evaluating treatment response, especially when CT scans are inconclusive, but its role remains uncertain [31]. These limitations of CA 19‐9 and PET‐CT underline the need for additional, more accessible prognostic tools. Our study suggests integrating ΔSMI with CA 19‐9 for a more comprehensive assessment in selecting candidates for LRTs. By incorporating ΔSMI, the precision of treatment planning can be enhanced and care better tailored to individual patients with pancreatic cancer.

In our study, chemotherapy response was identified as a significant prognostic factor, with post‐chemotherapy responses (PD vs. PR/SD) strongly correlating with both PFS and OS. However, the efficacy of conventional CT imaging in assessing treatment response is limited, especially PR, which is the key to evaluating whether to apply LRT [32, 33, 34, 35]. The primary limitation of post‐chemotherapy CT is its inability to accurately differentiate viable tumour cells from posttreatment fibrotic or inflammatory changes [35]. This emphasizes the need for more precise and reliable methods to evaluate treatment response in pancreatic cancer.

Our study had several limitations. First, its retrospective nature may introduce selection bias, potentially by not fully representing all patient scenarios. In particular, our analysis of four chemotherapy cycles as a treatment evaluation point may not capture the full spectrum of patient responses, potentially limiting the broader applicability of our findings. Second, the definition of early application of LRT as within 3 months post‐FOLFIRINOX chemotherapy limits the inclusion of patients who received LRT after this period, potentially affecting the generalizability of our findings. Third, our study focused exclusively on patients who underwent FOLFIRINOX therapy. This homogeneous patient population limits the applicability of our findings to those treated with different chemotherapy regimens or therapeutic approaches. Fourth, the reliance on conventional CT imaging for assessing post‐chemotherapy response has inherent limitations. Finally, while the prognostic significance of perichemotherapy ΔSMI and CA 19‐9 levels is promising, further validation in a broader patient cohort is necessary to enhance the generalizability and applicability in clinical practice.

In conclusion, our study identified progressive sarcopenic deterioration after four cycles of chemotherapy as associated with poor survival outcome in patients with BR or LAPC after FOLFIRINOX. This was the first investigation of the optimal clinical setting for the early application LRTs using the ΔSMI and post‐chemotherapy CA 19‐9.

## Conflicts of Interest

The authors declare no conflicts of interest.

## Supporting information


**Data S1** Supplementary information.
