# Supplementary material for: Skeletal Muscle Index Changes on Locoregional Treatment Application After FOLFIRINOX and Survival in Pancreatic Cancer
Source: J Cachexia Sarcopenia Muscle. 2024 Nov 23;16(1):e16343. doi: 10.1002/jcsm.13643 (PMC11670158; doi:10.1002/jcsm.13643)
Supplement: Supplementary file 1 — Data S1 Supplementary information. [file JCSM-16-e16343-s001.docx]

**Supporting Information S1. Resectability Assessment**

Resectability was determined according to the National Comprehensive Cancer Network (NCCN) guidelines.^1^ The borderline resectable pancreatic cancer (BRPC) was defined as either venous involvement of the superior mesenteric vein (SMV) or portal vein (PV) with narrowing or occlusion of the vein that allows resection and reconstruction of the vein, or encasement of the gastroduodenal artery with short segment encasement of the hepatic artery, and abutment of the superior mesenteric artery (SMA) that is less than 180° of the circumference of the vessel wall. The locally advanced pancreatic cancer (LAPC) was defined as unreconstructable SMV/PV involvement, or greater than 180° SMA encasement, celiac encasement, or inferior vena cava involvement. Treatment plan were determined by a multidisciplinary team.

**Supporting Information S2.** Flow chart of our study. PDAC, pancreatic ductal adenocarcinoma; FOLFIRINOX, folinic acid, 5-fluorouracil, irinotecan, and oxaliplatin; BRPC, borderline resectable pancreatic cancer; LAPC, locally advanced pancreatic cancer; LRT, loco-regional treatment


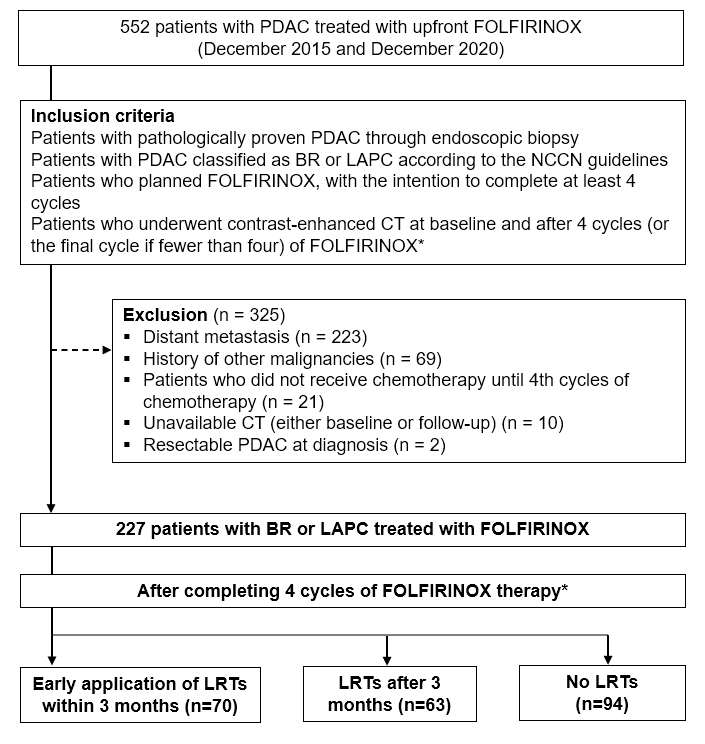


^*^ Two patients underwent surgery after completing 2 and 3 cycles of FOLFIRINOX, respectively.

**Supporting Information S3. Body Composition Analysis**

CT examinations were performed using multidetector CT scanners: SOMATOM Sensation 64, SOMATOM definition edge, and SOMATOM Definition Flash 128 (Siemens Healthineers), Toshiba Aquilion 64 detector (Toshiba Medical Systems), and LightSpeed VCT 64 or Discovery CT 750 (GE Healthcare). CT protocols were generally determined based on the physicians' clinical impression. For contrast-enhanced CT exams, 120 mL of nonionic iodinated contrast material was administered through the antecubital vein with a power injector at a rate of 3–4 mL/s. All CT exams included portal venous phase obtained at 70 s after the initiation of contrast injection. The scanning parameters were as follows: 100–120 kVp, 180–200 mAs, 2.5‒5 mm slice thickness, table speed of 26.5–39.4 mm per rotation (pitch, 0.83–1.07), and a single-breath hold helical acquisition time of 4–6 s.

Pre chemotherapy CT scans classed as prechemotherapy were performed at a median of 15 days (range, 1‒65 days) before 1^st^ chemotherapy, while post 4 cycles chemotherapy CT scans were performed at a median of 13 days (range, 1‒80 days) after 4 cycles of chemotherapy. The median time interval between prechemotherapy CT and post 4 cycles chemotherapy CT was 87 days (range, 75‒99 days).

Sarcopenia was assessed on abdominal CT using an artificial intelligence software (AID-UTM; iAID Inc.) developed using a fully convolutional network segmentation technique.^4^ A radiologist (14 years of experience in abdominal imaging) who was blinded to the clinical information selected an axial CT slice at the third lumbar vertebra (L3) inferior endplate level

in the pre-chemotherapy and post 4 cycles of chemotherapy CT images. The selected CT image was saved as a digital imaging and communications in medicine (DICOM) file. Then, the selected CT images were automatically segmented to create the boundaries of the entire abdominal muscle group (i.e., psoas, paraspinal, transversus abdominis, quadratus lumborum, rectus abdominis, quadriceps, and internal and external obliques), and the total abdominal muscles area (TMA) was measured. The skeletal muscle index (SMI) was calculate as TMA/height (cm^2^/m^2^).

**Supporting Information S4. Treatment**

During the study period, FOLFIRINOX or modified FOLFIRINOX defined as 2 weeks per standard or modified dose combination regimen comprising folinic acid, 5-fluorouracil, irinotecan, and oxaliplatin were preferred regimen in BRPC or LAPC patients with fit medical condition and an Eastern Cooperative Oncology Group (ECOG) performance status of 0 or 1. After the initiation of FOLFIRINOX, surgeon and/or physicians evaluated the resectability mainly based on patient condition and tumor extent observed on imaging studies, and then reevaluated it every four cycles while continuing FOLFIRINOX.

Surgeons and physicians maintained a proactive stance towards considering surgical resection for patients showing favorable initial responses to FOLFIRINOX treatment, regardless of their initial classification as BRPC or LAPC.

The use of RT for patients surgically eligible was not routine and was limited to the preference of certain surgeons or cases where there were concerns about complete resection. Additionlly, RT was recommended in unresectable patients, those intolerance to systemic therapy, cases with suspicious or confirmed localized progression, or severe tumor-related symptoms.

The method of RT was determined according to the tumor extent, with long course concurrent chemoradiotherpy using capecitabine or 5-FU for suspected or confirmed bowel invasion. Otherwise, five to ten fractions of short course RT alone with intensity modulation were used. Proton beam RT has been considered as the first option since 2017, especially in cases where the distance from the bowel is more than 0.5 to 1 cm for short course RT. More detailed information about RT was introduced in a previous articles.^2,3^

**Supporting Information S5. Characteristics of High and Low ΔSMI Groups**

|  | **High ΔSMI group**  **(n=114)** | **Low ΔSMI group**  **(n=113)** | ***P value*** |
| --- | --- | --- | --- |
| Age (year) | 60 (56, 67) | 61 (56, 67) | 0.951 |
| Sex, Male | 63 (55.3) | 61 (54.0) | 0.952 |
| BMI^*^ | 22.9 ± 2.9 | 23.1 ± 2.6 | 0.563 |
| Initial resectability |  |  | 0.389 |
| BR | 45 (39.5) | 52 (46.0) |  |
| LA | 69 (60.5) | 61 (54.0) |  |
| Chemotherapy response^†^ |  |  | 0.571 |
| PR | 55 (48.3) | 60 (53.1) |  |
| SD | 52 (45.6) | 49 (43.4) |  |
| PD | 7 (6.1) | 4 (3.5) |  |
| Baseline CA19-9 category^‡^ |  |  | 0.128 |
| < 500 U/ml | 72 (63.2) | 83 (73.5) |  |
| ≥ 500 U/ml | 42 (36.8) | 30 (26.5) |  |
| Post CTx CA19-9 category^‡^ |  |  | 0.027 |
| < 37 U/ml | 38 (33.3) | 55 (48.7) |  |
| ≥ 37 U/ml | 76 (66.7) | 58 (51.3) |  |
| Resected cases after FOLFIRINOX therapy | 34 (29.8) | 42 (37.2) | 0.302 |
| Resection margin (If resected) |  |  |  |
| R0 | 29 (85.3) | 37 (88.1) | 0.745 |
| R1*/*R2 | 5 (14.7) | 5 (11.9) |  |

Note_ Data are expressed as n (%) or median (interquartile range).

^*^ Mean ± standard deviation

^†^ At the time of completion of 4 cycles of chemotherapy

^‡^ The CA19-9 categories were categorized: Baseline (< 500 U/mL vs. ≥ 500 U/mL) and post-chemotherapy (< 37 U/mL [normalization either normal or elevated baseline CA 19-9] vs. ≥ 37 U/mL).

BMI, body mass index; BR, borderline resectable; CA19-9, carbohydrate antigen 19-9; CTx, chemotherapy; FOLFIRINOX, folinic acid, 5-fluorouracil, irinotecan, and oxaliplatin; LA, locally advanced; LRT, loco-regional treatment; PD, progressive disease; PR, partial response; SD, stable disease

**Supporting Information S6. Characteristics According to the Time of Loco-regional Treatments After Completing 4 Cycles of FOLFIRINOX**

|  | LRTs  within 3 months  (n = 70) | LRTs after 3 months  or Non-LRTs  (n = 157) | ***P value*** |
| --- | --- | --- | --- |
| Age (year) | 60 (55, 67) | 61 (56, 67) | 0.365 |
| Sex, Male | 34 (48.6) | 69 (44.0) | 0.616 |
| BMI^*^ | 23.3 ± 2.4 | 22.9 ± 2.9 | 0.258 |
| Initial resectability |  |  | <0.001 |
| BR | 49 (70.0) | 48 (30.6) |  |
| LA | 21 (30.0) | 109 (69.4) |  |
| Chemotherapy response^†^ |  |  | 0.092 |
| PR | 42 (60.0) | 73 (46.5) |  |
| SD | 27 (38.6) | 74 (47.1) |  |
| PD | 1 (1.4) | 10 (6.4) |  |
| Baseline CA19-9 category^‡^ |  |  | 0.017 |
| < 500 U/ml | 56 (80.0) | 99 (63.1) |  |
| ≥ 500 U/ml | 14 (20.0) | 58 (36.9) |  |
| Post CTx CA19-9 category^‡^ |  |  | <0.001 |
| < 37 U/ml | 41 (58.6) | 52 (33.1) |  |
| ≥ 37 U/ml | 29 (41.4) | 105 (66.9) |  |
| Resected cases after FOLFIRINOX therapy | 54 (77.1) | 22 (14.0) | <0.001 |
| If resected, resection margin |  |  | 0.265 |
| R0 | 45 (83.3) | 21 (95.4) |  |
| R1/R2 | 9 (16.7) | 1 (4.6) |  |

Note_ Data are expressed as n (%) or median (interquartile range) [range, minimum-maximum], unless otherwise indicated.

^*^ Mean ± standard deviation

^†^At the time of completion of 4 cycles of chemotherapy

^‡^ The CA19-9 categories were categorized: Baseline (< 500 U/mL vs. ≥ 500 U/mL) and post-chemotherapy (< 37 U/mL [normalization either normal or elevated baseline CA 19-9] vs. ≥ 37 U/mL).

BMI, body mass index; BR, borderline resectable; CA19-9, carbohydrate antigen 19-9; CTx, chemotherapy; FOLFIRINOX, folinic acid, 5-fluorouracil, irinotecan, and oxaliplatin; LA, locally advanced; LRT, loco-regional treatment; PD, progressive disease; PR, partial response; SD, stable disease

**Supporting Information S7. Characteristics According to the Group of ΔSMI and Post-chemotherapy CA 19-9**

|  | **No risk factor**  **(n = 55)** | **Any one risk factor**  **(n = 96)** | **Two risk factors**  **(n = 76)** | ***P value*** |
| --- | --- | --- | --- | --- |
| Age (year) | 61 (56, 68) | 60 (56, 67) | 60.5 (56, 66) | 0.808 |
| Sex, Male | 32 (58.2) | 52 (54.2) | 40 (52.6) | 0.814 |
| BMI^*^ | 23.0 ± 2.7 | 22.8 ± 2.6 | 23.2 ± 3.0 | 0.700 |
| Initial resectability |  |  |  | 0.028 |
| BR | 31 (56.4) | 41 (42.7) | 25 (32.9) |  |
| LA | 24 (43.6) | 55 (57.3) | 51 (67.1) |  |
| Chemotherapy response^†^ |  |  |  | 0.125 |
| PR | 32 (58.2) | 52 (54.2) | 31 (40.8) |  |
| SD | 22 (40.0) | 41 (42.7) | 38 (50.0) |  |
| PD | 1 (1.8) | 3 (3.1) | 7 (9.2) |  |
| Baseline CA19-9 category^‡^ |  |  |  | <0.001 |
| < 500 U/ml | 55 (100) | 65 (67.7) | 35 (46.1) |  |
| ≥ 500 U/ml | 0 (0) | 31 (32.3) | 41 (53.9) |  |
| Post CTx CA19-9 category^‡^ |  |  |  | <0.001 |
| < 37 U/ml | 55 (100) | 38 (39.6) | 0 (0) |  |
| ≥ 37 U/ml | 0 (0%) | 58 (60.4) | 76 (100) |  |
| Baseline PNI^§^ | 43.0  (41.0, 45.0) | 44.0  (42.0, 46.0) | 43.5  (41.0, 46.0) | 0.238 |
| Post CTx PNI^§^ | 41.0  (38.0, 43.5) | 41.0  (39.0, 43.0) | 40.0  (36.0, 42.0) | 0.024 |
| Baseline Sarcopenia, total | 8 (14.6) | 14 (14.6) | 8 (10.5) | 0.698 |
| Baseline Sarcopenia, male | 5 (15.6) | 7 (13.5) | 5 (12.5) | 0.895 |
| Baseline Sarcopenia, female | 3 (13.0) | 7 (15.9) | 3 (8.3) | 0.644 |
| Post CTx Sarcopenia, total | 10 (18.2) | 25 (26.0) | 26 (34.2) | 0.121 |
| Post CTx Sarcopenia, male | 7 (21.9) | 18 (34.6) | 17 (42.5) | 0.183 |
| Post CTx Sarcopenia, female | 3 (13.0) | 7 (15.9) | 9 (25) | 0.490 |
| Δ SMI (%), total^‡^ | -0.7  (-3.1, 2.1) | -3.8  (-10.9, 0.6) | -12.1  (-16.9, -9.2) | <0.001 |
| Δ SMI (%), male^‡^ | 2.4  (-4.7, -0.4) | -8.1  (-14.0, -3.0) | -14.1  (-17.9, -11.0) | <0.001 |
| Δ SMI (%), female^‡^ | 0.5  (-0.8, 5.1) | -0.6  (-4.9, 2.8) | -9.2  (-16.5, -6.7) | <0.001 |
| Δ SMI (%) category^‡^ |  |  |  | <0.001 |
| High SMI group | 0 (00) | 38 (39.6) | 76 (100) |  |
| Low SMI group | 55 (100) | 58 (60.4) | 0 (0) |  |
| Resected cases after FOLFIRINOX therapy | 25 (45.5) | 38 (39.6) | 13 (17.1) | <0.001 |
| If resected, resection margin |  |  |  | 0.109 |
| R0 | 22 (88.0) | 35 (92.1) | 9 (69.2) |  |
| R1/R2 | 3 (12.0) | 3 (7.9) | 4 (30.8) |  |

Note_ Data are expressed as n (%) or median (interquartile range) [range, minimum-maximum], unless otherwise indicated.

^*^ Mean ± standard deviation

^†^At the time of completion of 4 cycles of chemotherapy

^‡^ The CA19-9 categories were categorized: Baseline (< 500 U/mL vs. ≥ 500 U/mL) and post-chemotherapy (< 37 U/mL [normalization either normal or elevated baseline CA 19-9] vs. ≥ 37 U/mL).

^§^ The PNI was calculated as 10 × serum albumin (g/dl) + 0.005× total lymphocyte count (per mm^3^).

BMI, body mass index; BR, borderline resectable; CA19-9, carbohydrate antigen 19-9; CTx, chemotherapy; FOLFIRINOX, folinic acid, 5-fluorouracil, irinotecan, and oxaliplatin; LA, locally advanced; LRT, loco-regional treatment; PD, progressive disease; PNI, prognostic nutrition index; PR, partial response; SD, stable disease; SMI, skeletal muscle index

**REFERENCES**

1. National Comprehensive Cancer Network. Pancreatic Adenocarcinoma (Version 1.2022), www.nccn.org/professionals/ohysician_gls/pdf/pancreatic.pdf. Accesssed 15 Dec 2023.

2. Shin H, Yu JI, Park HC, Yoo GS, Cho S, Park JO, et al. The Feasibility of Stereotactic Body Proton Beam Therapy for Pancreatic Cancer. *Cancers (Basel)* 2022;**14**.

3. Kim K, Park HC, Yu JI, Park JO, Hong JY, Lee KT, et al. Impact and optimal timing of local therapy addition in borderline resectable or locally advanced pancreatic cancer after FOLFIRINOX chemotherapy. *Clin Transl Radiat Oncol* 2024;**45**:100732.

4. Park HJ, Shin Y, Park J, Kim H, Lee IS, Seo DW, et al. Development and Validation of a Deep Learning System for Segmentation of Abdominal Muscle and Fat on Computed Tomography. *Korean J Radiol* 2020;**21**:88-100.
